# Development of a Bladder Cancer-on-a-Chip Model to Assess Bladder Cancer Cell Invasiveness

**DOI:** 10.3390/cancers16152657

**Published:** 2024-07-26

**Authors:** Desiree J. Ewell, Nita Vue, Sakib M. Moinuddin, Tanoy Sarkar, Fakhrul Ahsan, Ruth L. Vinall

**Affiliations:** Department of Pharmaceutical & Biomedical Sciences, California Northstate University College of Pharmacy, Elk Grove, CA 95757, USA; desiree.ewell8628@cnsu.edu (D.J.E.); nita.vue8890@cnsu.edu (N.V.); sakib.moinuddin@cnsu.edu (S.M.M.); tanoy.sakar@cnsu.edu (T.S.); fakhrul.ahsan@cnsu.edu (F.A.)

**Keywords:** bladder cancer, organ-on-a-chip, invasion

## Abstract

**Simple Summary:**

The development and use of migrastatic drugs could help improve the outcomes for patients with high-grade non-muscle-invasive bladder cancer and muscle-invasive bladder cancer by inhibiting bladder cancer cell invasion and thereby decreasing the likelihood of disease progression and metastasis. A key barrier to this is the lack of suitable models to assess bladder cancer cell invasiveness. Here, we describe a bladder cancer-on-a-chip model. We demonstrate that our model supports the retention of the bladder cancer cell phenotype and can be used to reproducibly assess and quantify the invasiveness of live bladder cancer cells. Treatment with ATN-161, a well-known migrastatic drug, caused a dose-dependent decrease in bladder cancer cell invasiveness thereby validating the ability of our model to test migrastatic drugs. To our knowledge, a bladder cancer-on-a-chip model that can specifically assess bladder cancer invasiveness has not previously been described.

**Abstract:**

We have developed a bladder cancer-on-a-chip model which supports the 3D growth of cells and can be used to assess and quantify bladder cancer cell invasiveness in a physiologically appropriate environment. Three bladder cancer cell lines (T24, J82, and RT4) were resuspended in 50% Matrigel^®^ and grown within a multi-channel organ-on-a-chip system. The ability of live cells to invade across into an adjacent 50% Matrigel^®^-only channel was assessed over a 2-day period. Cell lines isolated from patients with high-grade bladder cancer (T24 and J82) invaded across into the Matrigel^®^-only channel at a much higher frequency compared to cells isolated from a patient with low-grade cancer (RT4) (*p* < 0.001). The T24 and J82 cells also invaded further distances into the Matrigel^®^-only channel compared to the RT4 cells (*p* < 0.001). The cell phenotype within the model was maintained as assessed by cell morphology and immunohistochemical analysis of E-cadherin. Treatment with ATN-161, an α5β1 integrin inhibitor and well-known migrastatic drug, caused a dose-dependent decrease in the invasiveness of the J82 cells (*p* < 0.01). The combined data demonstrate that our bladder cancer-on-a-chip model supports the retention of the bladder cancer cell phenotype and can be used to reproducibly assess and quantify the invasiveness of live bladder cancer cells.

## 1. Introduction

There is an urgent need to develop new treatment options for patients with advanced bladder cancer. The current 5-year survival rates for patients with invasive and metastatic bladder cancer are 39% and 8%, respectively [1]. Only ~50% of patients respond to cytotoxic chemotherapy-based regimens, and these therapies are associated with significant toxicity to patients [2,3]. Only ~20% of patients respond to immune checkpoint inhibitors [4,5].

Several migrastatic drugs, typically defined as drugs which inhibit cancer cell invasion and thereby delay or prevent metastasis, have been developed; however, the major focus remains on the development and use of cytotoxic chemotherapy and/or immunotherapy for the treatment of most cancers, including bladder cancer [6,7]. The fact that metastasis causes most solid cancer-related deaths supports more attention being given to this drug class; however, the efforts have been relatively limited. A major reason for this is the lack of availability of appropriate assays to assess cancer cell invasiveness.

During preclinical drug development, 2D assays, e.g., the Boyden chamber and scratch test assays, are typically used to assess the cancer cell invasiveness and response to treatments. These assays are of limited value as they do not allow for accurate modeling of in vivo conditions or drug delivery. Animal models, including patient-derived xenograft (PDX) models, can generate more robust data; however, they can be challenging, time consuming, and expensive to set up and work with, and PDX models only allow for the assessment of a relatively small number of patient specimens [8,9,10].

Organ-on-a-chip models can support the 3D culture of cells and recapitulation of tissue architecture and physiology. They can also support high-throughput drug screening studies and pharmacokinetic studies as well as the modeling of drug delivery [11,12,13,14]. Several organ-on-a-chip models have been developed to assess the cancer cell invasiveness, and some of these have been used to test migrastatic drugs [12,15,16]; however, to our knowledge, a bladder cancer-on-a-chip model which specifically assesses bladder cancer cell invasiveness has not been reported.

In addition to supporting the physiologically appropriate assessment of cellular processes, including invasiveness, organ-on-a-chip models can also be used to better understand the molecular mechanisms which drive disease progression and the mechanisms of drug resistance. Surprisingly little is known regarding the mechanisms which drive bladder cancer cell invasion into the muscle layer. Several associations with the expression of molecules which play a role in EMT and/or invasion in other cancers have been made [17,18,19,20]. For example, associations between bladder cancer progression and the decreased expression of E-cadherin [20], a key component of cell–cell adherens junctions, and alterations in the other molecules that play a role in EMT (e.g., Snail, Slug, Twist, and Zeb) have been reported [21,22,23]. The increased expression of matrix metalloproteinases (MMPs, e.g., MMP9) which can degrade the extracellular matrix (ECM) and thereby support invasion has also been reported and is associated with worse patient outcomes [24,25,26]. The exact mechanisms by which the expression and/or activity of these molecules are regulated and how they contribute to bladder cancer cell invasion remain to be fully elucidated.

## 2. Materials and Methods

### 2.1. Cell Lines and Culture

The MB49-GFP cell line was a generous gift from Dr. Christopher Lucchesi. The T24 and RT4 cells were purchased from ATCC (ATCC, Manassas, VA, USA). All the cell lines were maintained in RMPI1640 supplemented with 10% FBS as previously described [27].

### 2.2. Design and Fabrication of the Chips

**Figure 1:**
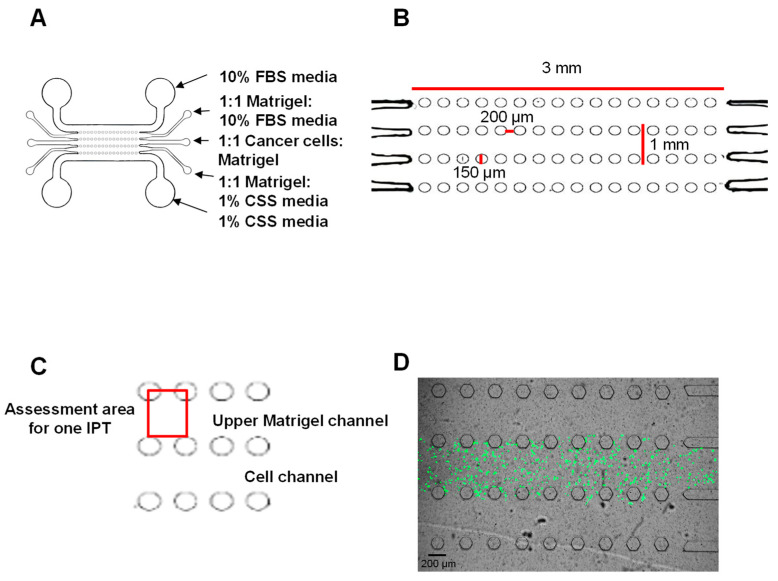
Microfluidic chip design and setup. Each chip has 5 horizontal channels, each of which is separated by 16 vertical posts (**A**). The distance between posts is 200 μm which is sufficient to allow for cell movement between the posts that separate the channels to occur (**B**). The central channel contains bladder cancer cells mixed 1:1 with Matrigel^®^, the 2 adjacent channels contain Matrigel^®^ mixed 1:1 with 10% FBS-containing media (upper channel) or 1:1 with 1% charcoal-stripped serum (CSS) media (lower channel), and the upper outer channel contains 10% FBS media, while the lower outer channel contains 1% CSS media (**A**). This setup creates a nutritional and chemotactic gradient to support directional invasion by cells towards the upper channels. Cell invasiveness is assessed by (1) counting the number of inter-post thresholds (IPTs) out of a total of 17 IPTs crossed by live cells invading from the central cell channel into the upper Matrigel^®^ channel, and (2) measuring furthest distance moved by cells into the Matrigel^®^ channel. (**C**) shows the measurement area for one IPT (red box). The bladder cancer-on-a-chip model was optimized using MB49 cells stably transfected with GFP (MB49-GFP). (**D**) shows a brightfield and fluorescence image overlay for MB49-GFP on day 0, i.e., the day of chip setup (green = MB49-GFP cells within the central channel).

The design and manufacture of the chips used for our bladder cancer-on-a-chip model have previously been described [28,29,30,31,32]; chips are generated through soft lithography. One exception to the manufacture process of chips for this study was that the chips were not coated with poly-D-lysine. This step was unnecessary because the cells in our bladder cancer-on-a-chip model are grown in Matrigel and do not need to adhere to the lower glass coverslip surface in any of the channels. Each chip has 5 channels that are 1 mm in diameter in width and 3 cm in length, and each channel accommodates a total volume of approximately 7 μL (Figure 1A,B). The channels are separated from each other by 16 posts meaning that there are a total of 17 spaces between the posts which separate the channels. Each post is 150 μm in diameter and the distance between each post is 200 μm (Figure 1B) which is sufficient to allow for the movement of the cells between the channels.

### 2.3. Introduction of the Cells and the Chip Setup

Bladder cancer cells were mixed 1:1 with Matrigel^®^ and RPMI media containing 1% charcoal-stripped serum (CSS) and loaded into the entry port for the central channel (Figure 1A,B). The chips were then placed in a humidified 100 mm Petri dish in a 37 °C tissue culture incubator for 60 min to allow for the cell/Matrigel^®^ to solidify. The upper channel that is adjacent to the central cell channel was then loaded with 1:1 Matrigel^®^ and RPMI1640 media containing 10% FBS, and the lower channel was loaded with 1:1 Matrigel^®^ and RPMI1640 media containing 1% CSS. The chips were again placed in a humidified 100 mm Petri dish in a 37 °C tissue culture incubator for 60 min to allow for the Matrigel^®^/media in the side channels to solidify. Lastly, the upper media channel was loaded with 10% FBS-containing RPMI1640 media, while the lower media channel was loaded with 1% CSS-containing RPMI1640 media. This setup creates a nutritional and chemotactic gradient and thereby supports the directional invasion by bladder cancer cells through the posts that separate the channels towards the upper Matrigel^®^ and media channels, i.e., the cells will preferentially invade towards the upper 10% FBS media-containing channels. Note that the charcoal stripping of serum reduces the levels of several molecules known to promote the invasiveness of bladder cancer cells, e.g., IGF-1 [33,34,35,36,37]; hence the use of the 1% CSS media within the lower channel helps further contribute to the generation of a chemotactic gradient within the chip. This setup models the in vivo environment in which cancer cells will preferentially invade in the direction of nearby blood vessels which are rich in nutrients and chemotactic factors. While the connection of the media channels to a microfluidic pump is possible using this chip model, a microfluidic pump was not used in this study, and instead, the media were replaced daily. The cell number per μL loaded into the central channel of the chip was optimized using the MB49-GFP cell line, and cell concentrations of 1000, 2000, and 5000 cells/μL were tested. The goal here was to determine what cell number could be accommodated within the central channel over a maximum 4-day period which would allow for the cell growth to be contained within the central channel, i.e., the cells would not be pushed into the adjacent Matrigel^®^ channels due to a lack of space in the central channel.

### 2.4. Assessment of Bladder Cancer Cell Invasion

For our initial experiments using MB49-GFP, the invasiveness of live cells was assessed on days 1, 2, and 4. For the subsequent experiments using the T24, J82, and RT4 cells, the invasiveness of live cells was assessed on days 1 and 2. The invasiveness of live bladder cancer cells was assessed in 2 ways in our model: (i) the assessment of the number of inter-post thresholds (IPTs) crossed out of a total of 17 IPTs on day 1 and day 2 of culture, and (ii) the maximum distance of invasion into the upper Matrigel^®^ channel. An IPT is defined as the area between 2 posts that separate the cell channel from the Matrigel^®^ channel. The metric assessed was ‘yes’ or ‘no’ as to whether any cells crossed each of the 17 IPTs that separated the cell and upper Matrigel^®^ channels on each chip to thereby give the number of IPTs crossed out of a possible 17 IPTs for each chip on day 1 and day 2. For measurement purposes, the horizontal IPT boundary was set at the outer edge of the posts between the cell channel and the Matrigel^®^ channel, and the vertical IPT boundary between 2 posts was set at the center of each post (Figure 1C). The furthest distance moved was calculated by measuring the distance from the center of posts to the cell which had invaded the furthest into the Matrigel^®^ channel. An Olympus IX83 fluorescent microscope (Olympus, Center Valley, PA, USA) and accompanying Leica Application Suite X software (version 3.7.5, Leica Microsystems, Wetzlar, Germany) were used to take images of each of the 17 IPT areas for each chip. The IPT boundaries were then superimposed onto these images and the ‘yes/no’ did any cells cross the IPT assessment performed. These same images were used to assess the furthest distance moved by the cells into the Matrigel^®^ layer.

### 2.5. Use of Adenovirus-GFP and a Quantum Dot-Based Tracker to Support the Cell Visualization

To determine whether the fluorescence labeling of the live cells within chips was possible, the J82 cells were either infected with adenovirus expressing GFP or incubated with a Quantum dot-based tracker (Qdots 655). Adenovirus expressing GFP was purchased from Vector Biolabs (Vector Biolabs, Malvern, PA, USA). The cells were infected with GFP-adenovirus per the manufacturer’s instructions prior to being mixed with Matrigel^®^ and introduced into the chip. Briefly, the cells were mixed with 50 MOI GFP-adenovirus and incubated at room temperature for 30 min before being centrifuged and resuspended in media, mixed 1:1 with Matrigel^®^, and then introduced into the chips. Qdots 655 were purchased from Thermo Fisher Scientific (Thermo Fisher Scientific, Waltham, MA, USA). The cells were incubated with Qdots 655 per the manufacturer’s instructions prior to being mixed with Matrigel^®^ and introduced into the chip. Briefly, the cells were incubated with the Qdots 655 for 1 h, then centrifuged and resuspended in media, mixed 1:1 with Matrigel^®^, and then introduced into the chips.

### 2.6. Immunofluorescence Analyses, Antibodies

For the immunofluorescence analyses, the chips were first washed with PBS and fixed with 4% paraformaldehyde; then a standard immunofluorescence staining protocol followed as previously described [29]. An Olympus IX83 fluorescent microscope (Olympus) and accompanying Leica Application Suite X software (Leica Microsystems) were used to capture the images. The cells were stained for E-cadherin (BD Transduction Laboratories, Franklin Lakes, NJ, USA cat #610182) and counterstained with DAPI (Vector Biolabs).

### 2.7. ATN-161 Treatments

ATN-161, an α5β1 integrin inhibitor and well-established migrastatic drug, was purchased from Selleck Chemicals (Selleck Chemicals, Houston, TX, USA). ATN-161 was added to the upper media channel of the chips on day 0 at concentrations of 0, 1, 10, and 100 μM, and the impact of the treatment on the invasiveness of live bladder cancer cells was assessed on days 1 and 2.

### 2.8. Statistical Analysis

At least three independent experiments were completed for each analysis described in this article. Data are shown as mean ± SD. A multiple group comparison was performed via one-way ANOVA followed by Tukey’s post hoc analysis using Prism software (version 9, Prism software, Irvine, CA, USA); *p* < 0.05 was considered statistically significant.

## 3. Results

### 3.1. Visualization of the Bladder Cancer Cells within the Chips: Cells Can Remain Viable within the Chips for at Least 10 Days

Figure 1A–C show the schematics of the bladder cancer-on-a-chip model. Our preliminary experiments to set up and optimize the bladder cancer-on-a-chip model were conducted using MB49 mouse bladder cancer cells stably transfected with GFP (MB49-GFP) (Figure 1D). MB49 is a well-established invasive bladder cancer cell line and shares many characteristics with human MIBC, e.g., cell surface markers and an immunological profile [38,39,40,41]. The MB49-GFP cells within the chips were visualized using brightfield as well as fluorescence microscopy on days 0, 1, 2, and 4. Figure 1D shows the MB49-GFP cells within a chip after being set up on day 0. A comparison of the brightfield and fluorescence images taken on days 0, 1, 2, and 4 confirmed that 100% of the MB49-GFP in the chips expressed GFP at these timepoints. The optimal number of MB49-GFP cells to load into a chip was found to be 5000 cells/μL. At this concentration of cells, the central cell channel of the chip was not completely filled with cells by day 4, i.e., there was still sufficient space for dividing cells to grow in the central channel and cells were not being forced into the adjacent Matrigel^®^ channels due to a lack of space. It is noteworthy that, as expected, the invasion by the cells was primarily observed in the upper Matrigel^®^ channel, i.e., the channel adjacent to the 10%

The FBS upper media channel and invasion by cells into the lower Matrigel^®^ channel, the channel adjacent to the 1% CSS media lower channel, was a rare event. This is due to the nutritional and chemotactic gradient created by adding 10% FBS media to the upper media channel versus 1% CSS media to the lower media channel of the chip.

**Figure 2:**
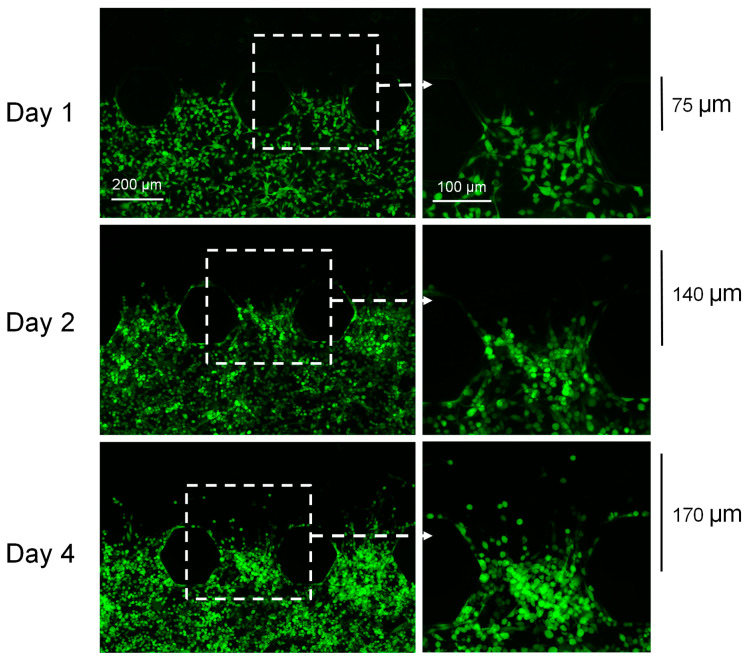
MB49-GFP bladder cancer cells can invade into the upper Matrigel^®^-containing channel as soon as one day after chip setup. By day 1, MB49-GFP cells had invaded up to 75 μm into the upper Matrigel channel^®^. This increased to 140 μm and 170 μm by days 2 and 4, respectively (green = MB49-GFP cells).

The MB49-GFP cells started to invade into the adjacent upper Matrigel^®^-containing channel as soon as day 1 (Figure 2). The MB49-GFP cells invaded a maximum distance of 75, 140, and 170 μm into the Matrigel^®^ channel by days 1, 2, and 4 of culture, respectively (Figure 2). An MTT assay, and the continued assessment of GFP expression, confirmed that the majority of the cells remained viable within the chips for up to 10 days. Figure S1 shows the MB49 cells in the chips on day 10 of culture before the MTT reagent was added (A) and 4 h after the addition of MTT (B). The majority of the cells stained blue 4 h after the addition of the MTT reagent indicating that the majority of the cells were still viable.

### 3.2. The Bladder Cancer-on-a-Chip Model Can Be Used to Assess and Quantify the Bladder Cancer Cell Invasiveness

**Figure 3:**
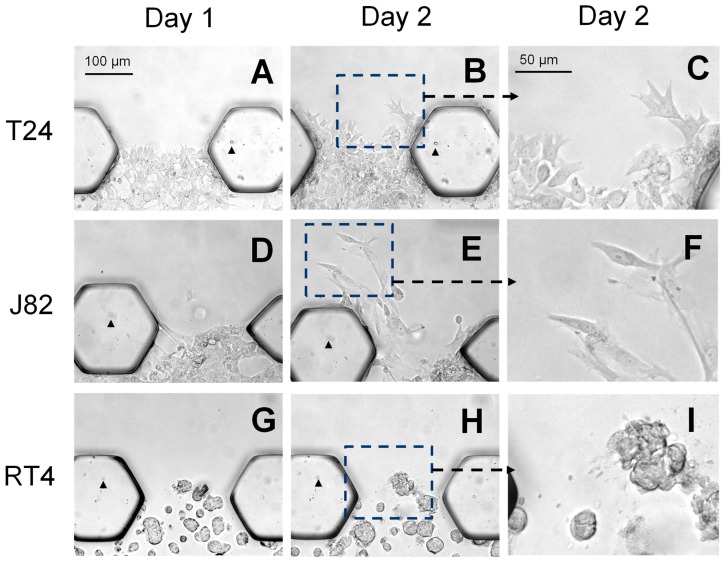
Cells derived from patients with high-grade bladder cancers maintained an invasive morphology within the chip model while cells derived from a low-grade bladder cancer maintained a non-invasive morphology. T24 and J82 cell lines, both of which were derived from patients with high-grade bladder cancer, extended cell processes in the direction of movement towards the upper channels ((**A**–**C**) and (**D**–**F**), respectively). In contrast, RT4 cells, which were derived from a patient with low-grade bladder cancer, remained rounded and retained a non-invasive morphology (**G**–**I**). Note that pictures of live cells were taken at the same position within the chip on days 1 and 2, which can be verified by observing unique markings on the chip posts (arrowheads) in (**A**,**B**). (T24 chip), (**D**,**E**). (J82 chip), and (**G**,**H**) (RT4 chip).

We subsequently used our chip model to assess the invasiveness of three human bladder cancer cell lines, T24, J82, and RT4. The invasiveness was assessed on days 1 and 2 based on our observation that a high level of invasion occurred as soon as day 1 when using the MB49-GFP cells (Figure 1D). This relatively short timeframe of assessment supports the further development of our chip model for high-throughput drug development and testing studies. Table 1 summarizes the known characteristics of the three human bladder cancer patient-derived cell lines: the J82 cell line was derived from a patient with high-grade MIBC (pT3, grade 3), the T24 cell line was derived from a patient with high-grade NMIBC (pTa, grade 3), and the RT4 cell line was derived from a patient with low-grade NMIBC (pT1, grade 1). The T24 and J82 cells invaded into the upper Matrigel^®^ channel as soon as day 1 and continued to invade further by day 2 (Figure 3A–C and Figure 3D–F, respectively). In contrast, the invasion by RT4 cells into the Matrigel^®^ channel was a rare event (Figure 3G–I). The T24 and J82 cells extended the cell processes outwards in the direction of the cell movement towards the upper media channel, and individual cells were observed invading into the Matrigel^®^ channel (Figure 3C,F), while the RT4 cells formed cell clusters and the cells remained rounded and did not extend the cell processes (Figure 3I). It is noteworthy that pictures of the live cells were taken at the same position within each chip on days 1 and 2. The representative images shown in Figure 3 help further emphasize the dramatic difference in the invasion rate of the T24 and J82 cells compared to the RT4 cell line. These data demonstrate that the relative invasiveness of the cell lines and cell phenotype as observed in our chip model aligns with the patient’s tumor grade: the grade 3 cancer cell lines, T24 and J82, were able to quickly invade into the Matrigel^®^ channel while the grade 1 cancer cell line, RT4, was not. T24 and J82 demonstrated an invasive morphology with extensive cell processes and the invasion by individual cells being observed. In contrast, the RT4 cells grew in clusters and did not extend the cell processes.

**Figure 4:**
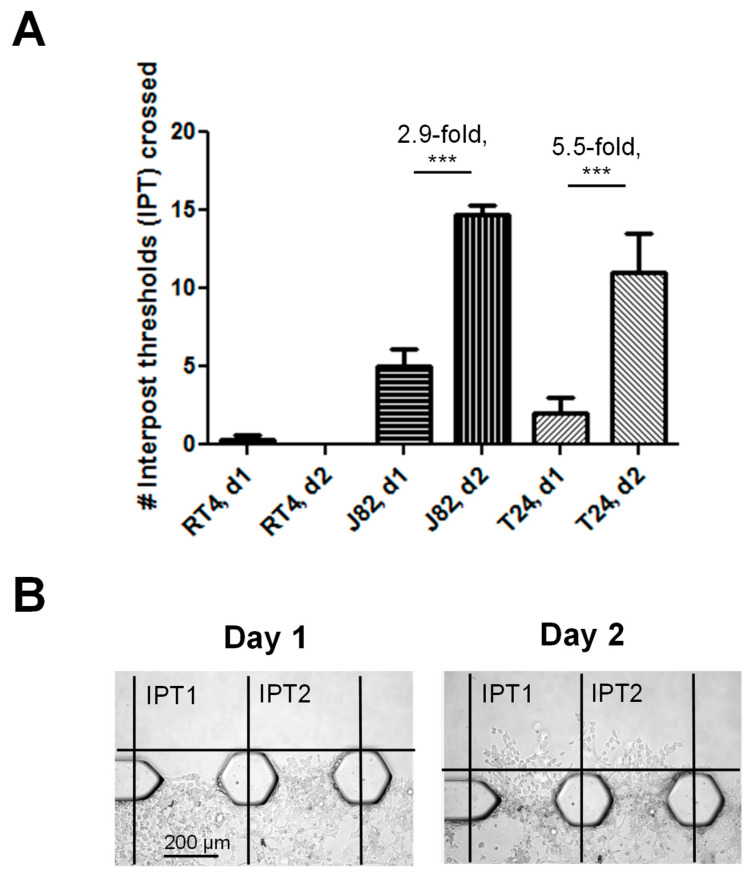
Assessment of number of inter-post thresholds (IPTs) that live bladder cancer cells are able to invade across by day 1 and day 2. A total of 16 posts separated channels within the chip from each other (Figure 1A–C) thereby creating a total of 17 spaces between channels, referred to as inter-post thresholds (IPTs), between which cells can invade across. As would be expected, the T24 and J82 cell lines, which were derived from patients with high-grade bladder cancer, were able to invade across multiple IPTs separating the cell channel and upper Matrigel^®^ channel as soon as day 1, while the RT4 cell line, which was derived from a patient with low-grade bladder cancer, rarely crossed any of the IPTs even by day 2 (**A**). Comparison of number of IPTs crossed on day 1 versus day 2 revealed a 2.9-fold and 5.5-fold increase in number of IPTs crossed for J82 and T24, respectively, thereby demonstrating the highly invasive nature of these bladder cancer cell lines. The boundary for IPT assessment was set between the outer edge of adjacent posts and the top of the upper Matrigel channel. (**B**) shows representative images that were used to assess whether or not an IPT had been crossed by J82 cells on day 1 and day 2 (‘#’ denotes IPTs which have been crossed by cells invading into the upper Matrigel^®^ channel). (*** = *p* < 0.0001).

**Figure 5:**
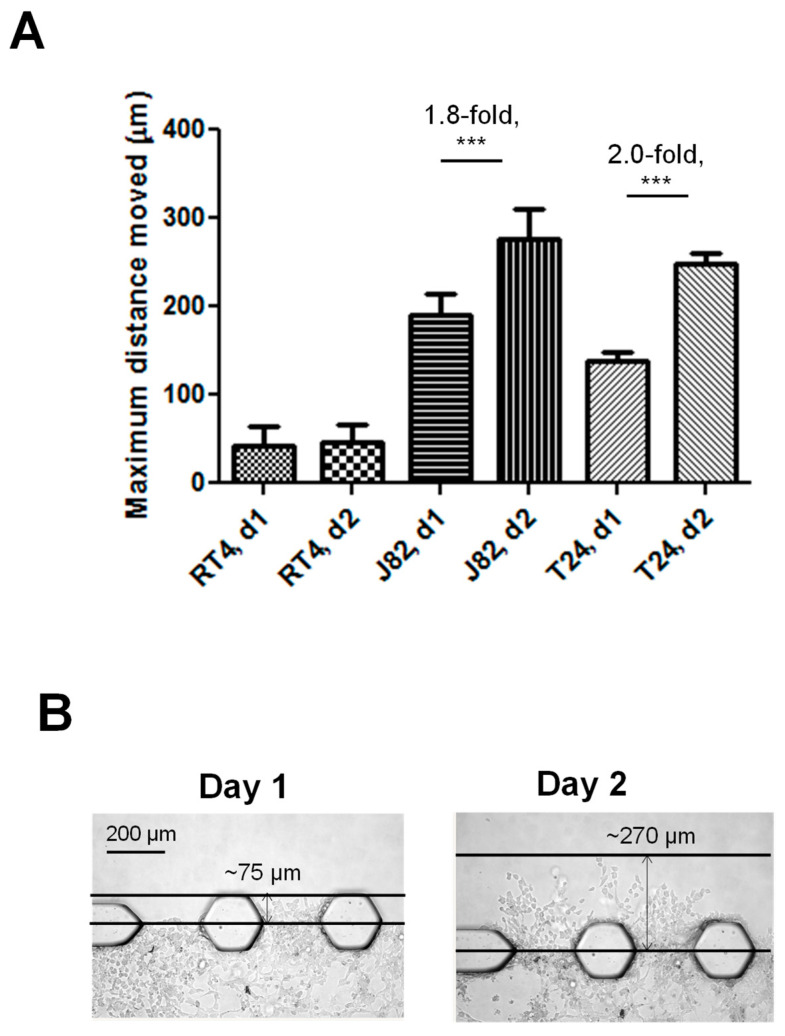
Assessment of maximum distance that live bladder cancer cells are able to invade into the upper Matrigel^®^ channel by days 1 and 2. Assessment of furthest distance moved by human bladder cancer cells further confirmed that bladder cancer cell phenotype is maintained within our chip model: as would be expected, the T24 and J82 bladder cancer cells lines, which were derived from patients with high-grade bladder cancer, invaded significant distances into the upper Matrigel channel, while the RT4 cells, which were derived from a patient with low-grade bladder cancer, rarely crossed IPT and invaded relatively short distances (**A**). (**B**) shows representative images that were used to assess furthest distance moved by T24 cells into the upper Matrigel channel. (*** = *p* < 0.0001).

The invasiveness of live bladder cancer cells was quantified in two ways: (i) the assessment of the number of inter-post thresholds (IPTs) crossed by the bladder cancer cells out of a total of 17 IPTs on day 1 and day 2 of culture (Figure 4), and (ii) the maximum distance moved by the cells into the Matrigel^®^ channel (Figure 5). An IPT is defined as the area between two posts that separate the cell channel from the Matrigel^®^ channel (Figure 1C). The metric assessed was ‘yes’ or ‘no’ as to whether any cells crossed the IPT for each of the 17 IPTs that separated the cell and Matrigel^®^ channels to thereby give the number of IPTs crossed out of a possible 17 IPTs for each chip. The IPT was set at the outer edge of the posts between the cell channel and the Matrigel^®^ channel, and representative images of the T24 cells crossing an IPT on day 1 and day 2 are shown in Figure 4B (‘#’ denotes an IPT that has been crossed). The furthest distance moved was calculated by measuring the distance from the center of the posts to the cell which had invaded the furthest into the Matrigel^®^ channel.

The number of IPTs crossed by the T24 and J82 cells on day 1 and on day 2 was significantly higher compared to the RT4 cells (Figure 4A, *p* < 0.0001). On day 1, an ~5.6-fold and ~15.6-fold difference in the number of IPTs crossed in the T24 and J82 chips, respectively, was observed compared to the RT4 chips. On day 2, this increased to an ~33.3-fold and ~48.0-fold difference. A comparison of day 1 and 2 data demonstrates that the T24 and J82 cells were able to continue to actively invade into the upper Matrigel^®^ channel over the 2-day period: a 2.9-fold increase and 4.7-fold increase in the number of IPTs crossed was observed for the T24 and J82 cell lines, respectively, by day 2 compared to day 1 (*p* < 0.0001). No statistically significant difference in terms of number of IPTs crossed on day 1 or day 2 was observed between the T24 and J82 cells, and this aligns with the fact that both were derived from patients with high-grade (grade 3) bladder cancer tumors.

A comparison of the bladder cancer cell invasiveness using the maximum distance moved metric demonstrated a similar trend as for the number of IPTs crossed: the T24 and J82 cells invaded significantly further distances into the Matrigel^®^ channel compared to the RT4 cells on both day 1 and day 2 (*p* < 0.0001) (Figure 5A). The average maximum distance moved into the Matrigel^®^ channel by the T24 and J82 cells by day 2 was 247.5 μm and 276.6 μm, respectively, while the average maximum distance moved by the RT4 cells was 46.7 μm. This equates to a 5.3-fold and 5.9-fold difference in the distance moved by the T24 and J82 cells, respectively, compared to the RT4 cells. The representative images of the T24 cells invading into the Matrigel^®^ channel are shown in Figure 5B. As with the IPT threshold data, a comparison of the d1 and d2 data supports that the T24 and J82 cells actively invaded into the upper Matrigel^®^ channel while the RT4 cells did not: 1.8-fold, 2.0-fold, and 1.5-fold increases in the maximum distance moved on day 2 compared to day 1 were observed for the T24 and J82 cells, and these increases were statistically significant (*p* < 0.0001). There was no increase in the maximum distance moved on d2 for the RT4 cells.

The combined data demonstrate that our model supports the recapitulation of the expected bladder cancer cell characteristics and morphology based on the grade of the patient’s tumor that they were derived from: the cell lines derived from high-grade patient tumors, T24 and J82, were able to invade a significant distance across multiple IPTs into the upper Matrigel^®^ channel while the cell line derived from a low-grade patient tumor, RT4, was not, and T24 and J82 maintained an invasive morphology while RT4 maintained a non-invasive morphology in the chip model. The combined data also demonstrate that both assessment measures of cell invasiveness, i.e., the number of IPTs crossed and the maximum distance moved, are reproducible and can be used to quantify the invasiveness of live bladder cancer cells over a 2-day period.

### 3.3. GFP-Adenovirus and Qdots Can Be Used to Further Support the Visualization of the Bladder Cancer Cells within the Chips

**Figure 6:**
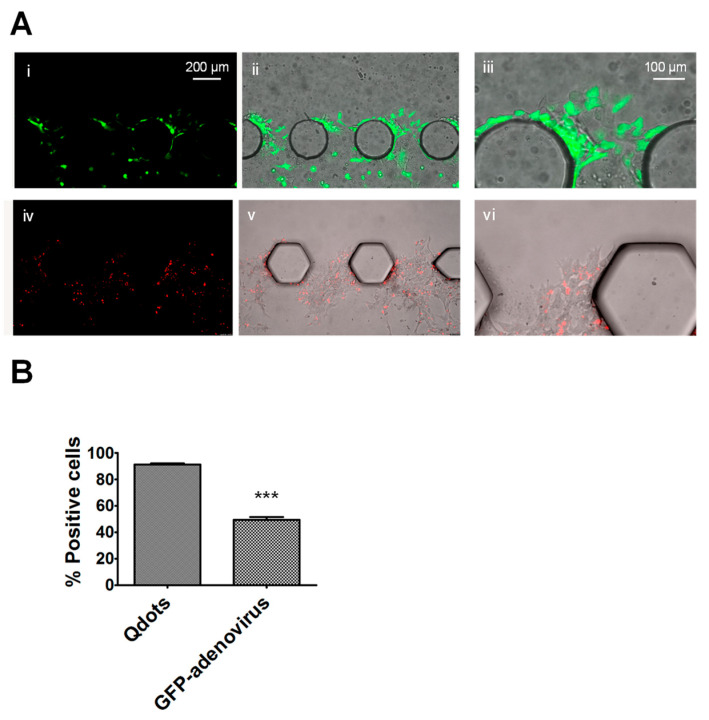
Infection of T24 bladder cancer cells with GFP-expressing virus or promoting uptake of fluorescent Qdot tracker prior to loading them into chips allows for visualization of cells using fluorescence microscopy. Infection of T24 cells with GFP-expressing adenovirus or Qdot tracker allowed cells to be visualized via fluorescence microscopy (**A**). (**A**(**i**),**A**(**iv**)) show fluorescence-only images of T24 cells infected with GFP-expressing adenovirus or T24 cells incubated with Qdot tracker, respectively. (**A**(**ii**),**A**(**v**)) show corresponding fluorescence and brightfield overlay images while (**A**(**iii**),**A**(**vi**)) show higher magnification overlay images. Comparison of representative brightfield and fluorescence images revealed an average 90.7% versus an average 49.3% positivity rate for T24 cells infected with GFP-expressing adenovirus versus T24 cells incubated with Qdot tracker (**B**). (*** = *p* < 0.0001).

While we demonstrate that the assessment and quantification of live bladder cancer cell invasion using brightfield microscopy on consecutive days is possible (Figure 3, Figure 4 and Figure 5), the assessment of the invasion by live bladder cancer cells stably transfected with GFP (Figure 2) using fluorescence microscopy generated clearer images compared to brightfield images because they did not pick up the background noise-created dust or imperfections on the chip surface. This made the quantification of invasiveness easier and faster. As it is not always feasible or desirable to stably transfect cells, e.g., primary cells isolated from patients, we investigated the possibility of using GFP-adenovirus infection (Figure 6A(i–iii)) and the treatment of cells with fluorescent Qdots (Figure 6A(iv–vi)) to allow for fluorescence imaging of the live cells within the chip model. A comparison of brightfield microscopy cell counts and the number of cells positive for fluorescence labeling demonstrated that the average positivity rates for the live T24 cells infected with GFP-adenovirus were much higher compared to the live cells treated with Qdots (90.7% and 49.3%, respectively). In addition to the lower positivity rate with Qdots, another concern was that the Qdots were not evenly distributed throughout the cells and were often absent from the cell processes. This made it challenging to determine if invasion across the IPT had occurred and challenging to determine the maximum distance moved. While the positivity rate of GFP-adenovirus-infected cells was much higher compared to the Qdot-treated cells (90.7% vs. 49.3%), and the GFP fluorescence did extend into the cell processes, a concern is that this lower than 100% positivity rate could impact the accuracy of the assessments of bladder cancer cell invasion. To address this concern, GFP-adenovirus positivity would need to be determined for each experiment to allow comparisons to be made between the treatment groups and between repeat experiments.

### 3.4. Immunofluorescence Analyses Confirmed That E-Cadherin Expression Is Lower in Muscle-Invasive Bladder Cancer Cells Compared to Non-Muscle-Invasive Bladder Cancer Cells

**Figure 7:**
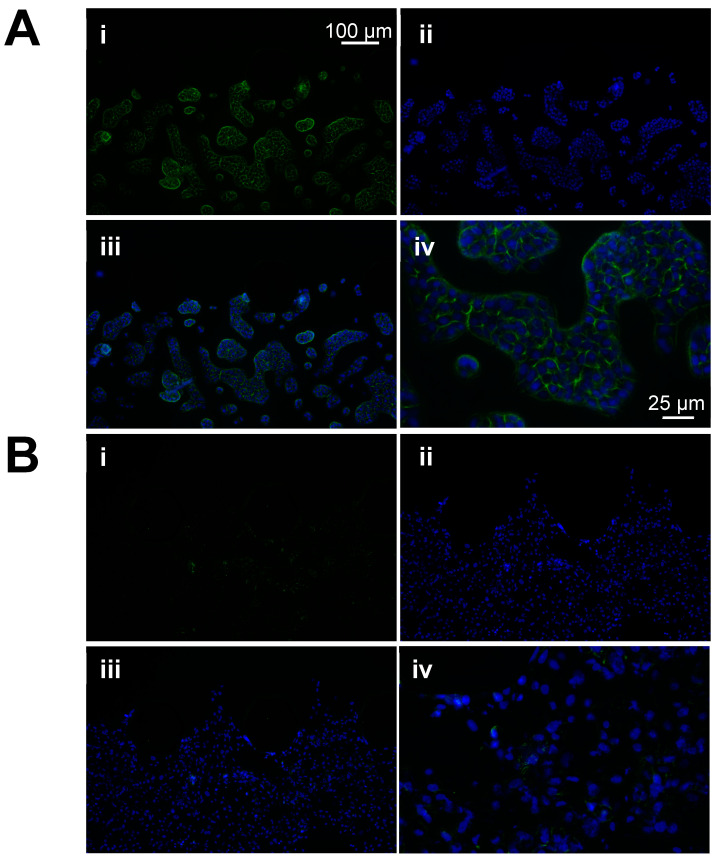
Immunofluorescence analysis of E-cadherin with chips. Staining of RT4 and J82 bladder cancer cells for E-cadherin demonstrated that immunofluorescence labeling analysis of cells within our chip model is possible (**A**,**B**). It also further confirmed that expected bladder cancer cell phenotype is maintained with the chip model: as expected, RT4 cells, which were derived from a patient with low-grade bladder cancer, expressed high levels of E-cadherin, a cell–cell junction protein, and expression was observed as a continuous layer along the outer edges of the cells (**A**). In contrast, J82 cells, which were derived from a patient with high-grade bladder cancer, expressed very low levels of E-cadherin (**B**). Decreased expression of E-cadherin is associated with epithelial mesenchymal transition (EMT) and metastasis. (Green = E-cadherin, blue = DAPI (cell nucleus), (**A**(**i**),**B**(**i**)) = Cadherin only, (**A**(**ii**),**B**(**ii**)) = DAPI only, (**A**(**iii**),**B**(**iii**)) = E-cadherin and DAPI overlay, (**A**(**iv**),**B**(**iv**)) = high magnification E-cadherin and DAPI overlay.

Other groups have established that the immunofluorescence analysis of specific molecules is possible within organ-on-a-chip models [42]. We confirmed this in our bladder cancer-on-a-chip model through immunofluorescence staining for E-cadherin and the use of a DAPI counterstain (Figure 7). E-cadherin is a cell–cell junction component which functions as a tumor suppressor in many cancers, including bladder cancer [43]. The decreased expression of E-cadherin is associated with epithelial mesenchymal transition (EMT) and is thought to promote metastasis [44,45]. Several studies have reported that a decreased expression of E-cadherin is associated with a higher risk of muscle-invasive bladder cancer in patients and worse survival rates [20,46]. In alignment with this, the RT4 cells, which were derived from a patient with low-grade NMIBC, expressed high levels of E-cadherin (Figure 7A(i,iii,iv)) while the J82 cells, which were derived from a patient with high-grade MIBC, expressed very lower levels of E-cadherin (Figure 7B(i,iii,iv)). In the RT4 cells, E-cadherin expression was observed as a more or less continuous layer around cells within cell clusters. In the J82 cells, E-cadherin was limited in expression to small areas between adjacent cells and was not expressed by all the cells. These data demonstrate that our bladder cancer-on-a-chip model can support the investigation of the molecular mechanisms which drive bladder cancer progression and chemoresistance.

### 3.5. Treatment of the J82 Cells with ATN-161, an α5β1 Integrin Inhibitor, Causes a Dose-Dependent Decrease in the Cell Invasiveness

**Figure 8:**
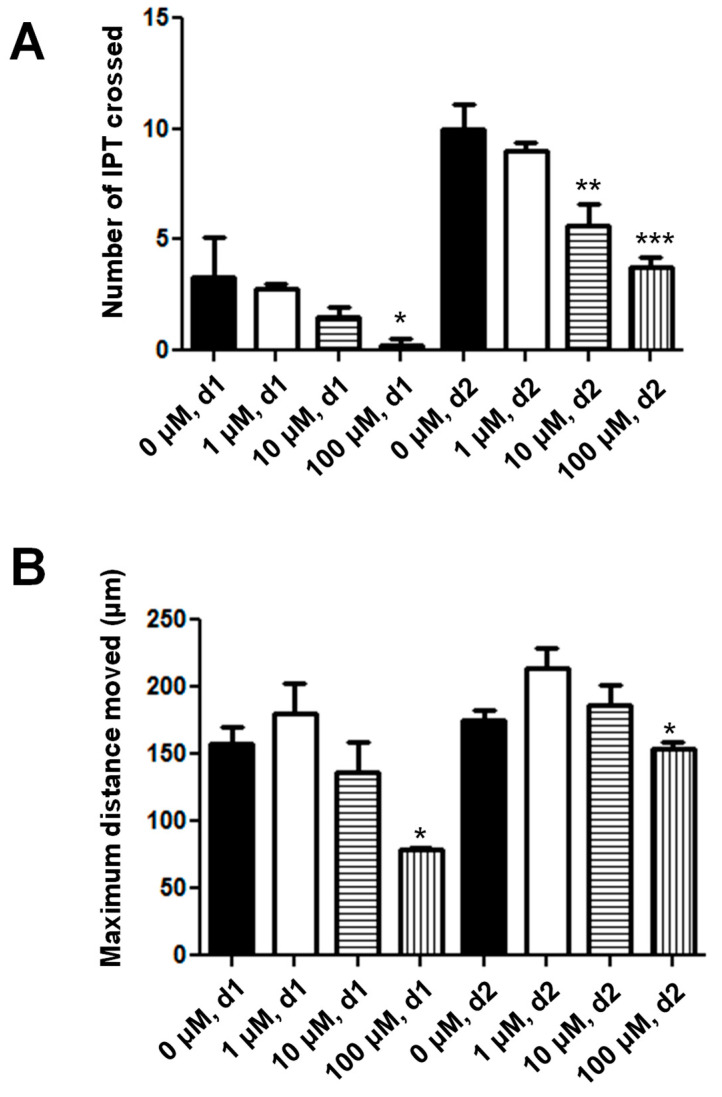
Treatment of J82 cells with ATN-161, an α5β1 integrin inhibitor, causes a dose-dependent decrease in J82 bladder cancer cell invasiveness. Treatment of J82 cells with ATN-161, a well-established migrastatic drug, caused a dose-dependent decrease in J82 bladder cancer cell invasiveness. Statistically significant decreases in both measures of bladder cancer cell invasiveness, i.e., number of IPTs crossed by live cells on days 1 and 2 (**A**) and furthest distance moved by live cells on days 1 and 2 (**B**), were observed following treatment with ATN-161. These data further confirm that expected bladder cancer cell behavior is maintained within our chip model and support usage of our chip for testing of other migrastatic drugs. (* = *p* < 0.01, ** = *p* < 0.001, *** = *p* < 0.0001).

Treatment of the J82 cells with ATN-161, an α5β1 integrin inhibitor and well-established migrastatic drug, resulted in a dose-dependent decrease in the number of IPTs crossed and the maximum distance moved on both day 1 and day 2 following treatment (Figure 8A,B). The highest dose of ATN-161, 100 μM, caused an ~13.0-fold and ~2.5-fold decrease in the number of IPTs crossed on day 1 (*p* < 0.01) and day 2 (*p* < 0.0001), respectively (Figure 8A), and an ~2-fold and ~1.1-fold decrease in the maximum distance moved on day 1 (*p* < 0.01) and day 2 (*p* < 0.01), respectively (Figure 8B). Other groups have reported that α5β1 integrin is expressed at high levels by J82 cells, and an association between increased α5β1 integrin and bladder cancer patient progression is known to exist [17,18,19]. Based on this, the ability of ATN-161 to decrease J82 invasiveness is expected and these data thereby help further demonstrate that the bladder cancer cell phenotype and behavior are retained in our bladder cancer-on-a-chip model and support its usefulness for the testing of migrastatic drugs.

## 4. Discussion

While multiple organ-on-a-chip models have been developed and described, the majority of these have focused on assessing the cancer cell proliferation and/or viability, typically in the context of the testing of cytotoxic drugs [47,48,49,50,51,52,53,54]. We have developed a bladder cancer-on-a-chip model that can be used to assess and quantify the bladder cancer cell invasiveness. This is an important metric to consider when determining the likelihood of cancer progression and when developing and testing migrastatic drugs as there are strong associations between cancer cell invasion and worse patient outcomes [55,56]. Our chip model supports the assessment of live bladder cancer cell invasion within a 3D model over a 2-day period. To our knowledge, this is the first bladder cancer-on-a-chip model developed to directly assess and quantify the bladder cancer cell invasion.

Multiple groups have published data supporting the need to develop and use 3D culture models to replace the current 2D models used for the functional assessment of cancer cell behaviors, including cancer cell invasion [57,58,59]. It is well accepted that 2D models are not representative of the in vivo conditions and that their use in preclinical research studies can result in false assumptions being made when designing subsequent animal studies and human clinical trials [8,60,61]. The use of a 3D culture system is particularly important for the assessment of invasion to help recapitulate the movement of cancer cells through the complex extracellular matrix (ECM) that is found within tissue and organ structures and to model the interplay that occurs between invading cells and the ECM [62,63,64]. In this study, we demonstrate that an organ-on-a-chip model can be used to assess and quantify the ability of bladder cancer cells to invade into an adjacent Matrigel^®^ layer. Matrigel^®^ is a solubilized basement membrane preparation extracted from the Engelbreth-Holm-Swarm (EHS) mouse sarcoma that is widely used to help recreate the tumor microenvironment within both in vivo and in vitro models [65]. Importantly, we demonstrate that our bladder cancer-on-a-chip model supports the retention of the bladder cancer cell phenotype and behaviors: bladder cancer cell lines derived from high-grade patient tumors were shown to be highly invasive within our model and invaded significant distances into the adjacent Matrigel^®^ channel over the 2-day timeframe. As expected, these cells exhibited a highly invasive morphology and extended cellular processes in the direction of cell movement. We demonstrate that immunostaining is possible in our model and staining for E-cadherin revealed that the invasive cells expressed very low levels of E-cadherin, a well-accepted hallmark of EMT and transition to an invasive phenotype. In contrast, RT4, the cell line derived from a low-grade tumor, was shown to be non-invasive within our model, and these cells were not able to invade into the Matrigel^®^ channel. These cells exhibited a cell morphology that is consistent with non-invasive bladder cells and expressed high levels of E-cadherin. Again, this is expected and helps further validate our model.

It is noteworthy that one of the bladder cancer cell lines that was found to be highly invasive in our model, the T24 cell line, was derived from a patient with high-grade NMIBC. A high tumor grade is associated with cancer progression, and patients with high-grade NMIBC are typically treated more aggressively because of this; however, the treatment decisions for these patients can be challenging [66]. It is possible that our model could be further developed and used for the functional assessment of the likelihood of the progression of high-grade NMIBC to MIBC and to thereby help further guide the treatment decisions for patients with high-grade NMIBC. In support of this, organ-on-a-chip models have been used to predict the patient response for other cancer types [67,68]. An important step will be to compare the ability of our model to predict the disease progression with metrics (e.g., cancer stage and grade), imaging data, and nomograms that are currently used to predict the disease progression [69,70,71]. It is noteworthy that a systematic review by Woo et al. demonstrated that MRI could predict the progression to MIBC with a sensitivity and specificity of up to 0.94 (95% CI 0.89–1.00) and 0.95 (95% CI 0.89–0.098), respectively [72]. The cost and test turnaround time of a chip-based test compared to these other methods will also be important factors to consider.

Several other 3D bladder cancer-on-a-chip models have been described; however, to our knowledge, none of these have been used to assess and quantify bladder cancer cell invasion. Liu et al. developed a bladder cancer-on-a-chip model which incorporated four cells types, including bladder cancer cells, and demonstrated that the expected phenotype of each cell type was maintained within their bladder cancer-on-a-chip model [52]. The physiological metrics assessed included cell proliferation and cell death. Xu et al. developed a bladder cancer-on-a-chip model to better understand the mitochondrial dysfunction in bladder cancer cells [73]. Kim et al. used bioprinting to develop a bladder cancer-on-a-chip model to assess the impact of drug treatments on cell proliferation and to assess the impact of drugs on cell–cell and cell–matrix junction numbers [53]. More recently, Kim et al. used this same model to assess the cell viability and proliferation in response to Bacillus Calmette–Guerin (BCG) [48]. While this group did not assess the invasiveness of bladder cancer cells in this study, they did assess the ability of differentiated THP-1 monocytes to invade into the bladder cancer cell layer.

Several organ-on-a-chip models have been developed to assess the cancer cell invasiveness for other cancer types [12,15,16]. For example, Truong et al. developed a breast cancer-on-a-chip model and demonstrated that the coculture of breast cancer cells with cancer-associated fibroblasts increased their invasiveness through a mechanism that involved glycoprotein nonmetastatic B [74]. Ozer et al. recently described the use of the commercially available OrganoPlate (Mimetas) organ-on-a-chip system to screen for drugs that would inhibit breast cancer cell invasion and intravasation [13]. This group used GSK429286, a Rho-kinase inhibitor and well-established migrastatic drug, as a positive control and demonstrated that it could inhibit the invasion and intravasation by MDA-MB-231 breast cancer cells in their model [13]. We have also used our bladder cancer-on-a-chip model to assess and quantify the impact of a migrastatic drug on cell invasiveness: treatment of bladder cancer cells with ATN-161, an α5β1 integrin inhibitor, caused a dose-dependent decrease in both of our measures of invasiveness. We chose to focus on the inhibition of the α5β1 integrin because α5β1 integrin is known to be overexpressed in MIBC tumors, and a high expression is associated with the progression to metastatic disease [17,18,19]. This, combined with our data, indicates that further investigation of the use of α5β1 integrin inhibitors for the treatment of MIBC and high-grade NMIBC is warranted.

Unlike our model, many of the chip models described above integrate multiple cell types. This is certainly important for mechanistic studies, and we plan to further develop our model to incorporate bladder cancer-associated fibroblasts, smooth muscle cells, and immune cells so that it can be used to help elucidate the mechanisms which drive bladder cancer cell invasion and to better understand how currently available migrastatic drugs affect these pathways. Our demonstration that bladder cancer cells can be labeled through infection with GFP-adenovirus will help support these studies because the visualization of live invading cells into channels containing other cell types will be possible. While our current model is somewhat simplistic in nature and does not include other cell types, a key benefit of this is that it is relatively quick and straightforward to set up, thereby making it well suited for the high-throughput testing of migrastatic drugs for the treatment of high-grade NMIBC and MIBC. In future studies, we will connect our chip model to microfluidic pumps to further support the recapitulation of in vivo conditions as well as to support pre-clinical pharmacokinetic drug studies.

## 5. Conclusions

In summary, we demonstrate that our bladder-on-a-chip model supports the retention of the expected bladder cancer cell phenotype and can successfully be used to assess and quantify the invasiveness of live bladder cancer cells. Our combined data support the usage of our model for the development and testing of migrastatic drugs for the treatment of bladder cancer. Lastly, we demonstrate that the immunostaining of the cells within chips is possible and this, as well as further model development, will allow our model to be used to further elucidate the molecular pathways which drive bladder cancer cell invasion.

## Figures and Tables

**Table 1:** Human bladder cancer cell line characteristics.

Cell Line	Stage	Grade	Notes
T24, NMIBC	pTa	G3	Female
J82, MIBC	pT3	G3	Male
RT4, NMIBC	pT1	G1	Male

## Data Availability

Primary data can be made available by request to the corresponding author.

## Supplementary Materials

The following supporting information can be downloaded at https://www.mdpi.com/article/10.3390/cancers16152657/s1, Figure S1: Bladder cancer cells can survive within chips for up to 10 days.
