# Supplementary material for: Development of a Bladder Cancer-on-a-Chip Model to Assess Bladder Cancer Cell Invasiveness
Source: Cancers (Basel). 2024 Jul 26;16(15):2657. doi: 10.3390/cancers16152657 (PMC11311651; doi:10.3390/cancers16152657)
Supplement: Supplementary file 1 [file cancers-16-02657-s001.zip › cancers-3089053-supplementary.pdf]

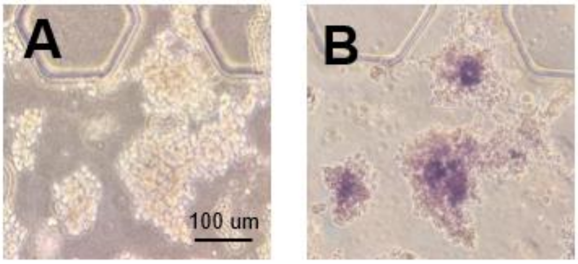

Supplementary Figure 1: Bladder cancer cells can survive within chips for up to 10 days. MTT assay analyses conducted using chips demonstrated that the majority of cells were still viable on day 10 (blue color indicates that cells contain active mitochondria and are alive). A = cells prior to addition of MTT reagent, B = cells incubated with MTT reagent for 3 hours.
